# Sovereign Credit Default Swap and Stock Markets in Central and Eastern European Countries: Are Feedback Effects at Work?

**DOI:** 10.3390/e22030338

**Published:** 2020-03-16

**Authors:** Sorin Gabriel Anton, Anca Elena Afloarei Nucu

**Affiliations:** Finance, Money, and Public Administration Department, Faculty of Economics and Business Administration, Alexandru Ioan Cuza University of Iasi, Carol I Avenue, No. 11, 700505 Iasi, Romania; anca.afloarei.nucu@uaic.ro

**Keywords:** CEE countries, credit default swap, stock market, vector autoregressive (VAR) model

## Abstract

The purpose of the paper is to investigate the relationship between sovereign Credit Default Swap (CDS) and stock markets in nine emerging economies from Central and Eastern Europe (CEE), using daily data over the period January 2008–April 2018. The analysis deploys a Vector Autoregressive model, focusing on the direction of Granger causality between the credit and stock markets. We find evidence of the presence of bidirectional feedback between sovereign CDS and stock markets in CEE countries. The results highlight a transfer entropy of risk from the private to public sector over the whole period and respectively, from the public to private transfer entropy of risk during the European sovereign debt crisis only in Romania and Slovenia. Another finding that deserves particular attention is that the linkage between the CDS spreads and stock markets is time-varying and subject to regime shifts, depending on global financial conditions, such as the sovereign debt crisis. By providing insights on the inter-temporal causality of the comovements of the CDS–stock markets, the paper has significant practical implications for risk management practices and regulatory policies, under different market conditions of European emerging economies.

## 1. Introduction

Over the past twenty years, the Credit Default Swap (CDS) market has experienced significant changes in terms of size and structure. Since its inception in the early 1990s, the size of the market increased rapidly, reaching the highest value in December 2007, when the notional amount outstanding for Over-The-Counter (OTC) CDS was $61.2 trillion. However, ten years later, the notional amount outstanding of CDS fell markedly to $9.4 trillion. On the other hand, there are significant changes in the structure of the market. For example, the share of CDS on sovereign entities has risen from 3.4% of the market in mid-2007 to 16% at the end of 2017 [1]. In light of this development, Bannier et al. [2] highlighted that CDS became “a leading indicator of corporate and sovereign entities’ default risk”.

Despite the numerous studies on the evolution and role of the CDS market conducted in the last ten years, little is known about the relationship between the CDS spreads and stock markets. Our paper is motivated by this gap in the extant literature dedicated to CDSs markets and by the need to provide better guidance to investors and/or policymakers in forecasting and monitoring stock market performance and/or CDS spreads in emerging economies. The aim of the paper is to analyze the dynamic relationship between the stock market and the sovereign CDS market on a sample of emerging economies from Central and Eastern Europe. The paper aims to offer insights regarding the transfer entropy between CDS and stock markets. We focus on information flow, analyzing, which the market takes the leadership in price discovery. In this paper, the concept of “transfer entropy” is used to describe the information flow in two financial markets.

The features of Central and Eastern Europe (CEE) countries make them a good framework to set up our analysis. These countries are characterized by different levels of economic and financial development, respectively various degrees of integration and political stability. The levels of sovereign default risk vary across time and counties, as highlighted by CDS premium and equity prices are different for each country. In line with Ters and Urban [3], we employ the credit default swaps as a measure for the credit risk to interpret the dynamics in sovereign risk. The transaction costs, liquidity level and the influences of the fiscal policies and budget deficits on sovereign CDS premium and equity prices are different [4] and these aspects motivate a country by country analysis.

The CDS–stock market relationship is drawing increasing attention since markets were exposed to crisis and investors have changed the perception and way of pricing of the sovereign credit risk. Following Tolikas and Tapaloglou [5], the empirical analysis answers to the following concerns: (i) which market reflects credit risk information first? and (ii) can we distinguish differences in the lead–lag relationship between the sovereign CDS and stock markets across economies and timeperiods? Our paper makes several contributions to the extant literature. First, it brings new insights into the lead–lag relationship between sovereign CDS spread and stock markets on a wide sample of European emerging countries. From the research perspective, it is interesting to understand which market leads the price discovery process for sovereign credit risk and the direction of information flow between stock markets and CDS in emerging countries. The relationship between the sovereign CDS market and the stock market has received little attention in Central and Eastern European (CEE) countries, only Bulgaria, Hungary and Poland being analyzed until May 2012 [4]. More specifically, we employ a large dataset of nine European emerging countries, our analysis covering a longer time period (i.e., January 2008 to April 2018) compared to the previous studies. Secondly, the global financial crisis of 2007 and the sovereign debt crisis provide us with a perfect testing ground for the entropy risk transfer between the markets. This is important because the relationship between the CDS spreads and stock markets may be time-varying and related to regime shifts, as highlighted by Shahzad et al. [6], on the example of the US. We want to test if this is applicable for European emerging economies as well, by looking at the behavior of the two markets on various subsamples of data. Further, as a new contribution, we investigate the effect of the permanent ban on outright short-selling of sovereign CDS contracts (the introduction of EU regulation 236/2012) on the lead–lag relationship between sovereign CDS and stock indices. The literature provides limited empirical evidence regarding the effects on price discovery effects after the adoption of the naked CDS ban [7]. We examine which market reacts faster in pricing the default risk or whether the price discovery tends to alternate between markets, after the ban on outright short selling of sovereign CDS in the euro area, which came into force in November 2012. The empirical results reveal that the stock market leads the price discovery for most countries.

The rest of the paper is organized as follows. Section 2 presents the literature review related to the CDS and stock market relationship. Section 3 describes the data and presents the econometric framework. Section 4 discusses the empirical results, while Section 5 concludes the paper and provides practical implications.

## 2. Literature Review

The review of the literature highlight that the linkage between the CDS spreads and stock markets have been documented at the microlevel, using firm-level data, and respectively at the macrolevel. Regarding the first strand, there is a large body of literature that investigates the relationship between CDS spreads and the stock market at the corporate level. Most of the studies have been performed on developed countries (see, inter alia, [8,9]) and only a few on the example of emerging markets [10].

The second strand of the literature can be divided into two groups: the first group studies the relationship between the CDS spread and the stock market at the sectoral level, while the second group at the country level. A lot of studies have been dedicated to the relationship between the CDS index spreads and the stock market at the sector level and most of them have been performed on the example of the United States [6,11,12,13,14]. In a recent work, Shahzad et al. [11] analyzed the causal linkages between ten U.S. credit default swaps and their corresponding stock sectoral markets, employing the quantile-on-quantile methodology and the nonparametric causality-in-quantiles tests. The authors report a negative and asymmetric nexus between the CDS and stock markets across the quantiles and a bidirectional Granger causality at the sectorial level. Shahzad et al. [6] examined the relationship between U.S. industry-wise credits and stock markets, using bootstrap Granger causality, over the period 2007–2014, and found that all stock markets Granger cause their CDS counterparts and there is also bidirectional causality for some industries (banking, healthcare and material industries). Lim et al. [12] use the transfer entropy approach to analyze the information flow between CDS and the stock market, at the sectorial level, in the US, over the period 2005–2012. The authors reveal a lead–lag relation between stock and the CDS market and found that modifications in transfer entropy for the CDS market precede those of the stock market.

Narayan [13] documented the dynamic relationship between sectoral equity returns and CDS spread returns in the U.S., using a Vector Autoregressive (VAR) methodology with data spanning the period February 2004–March 2012, and found that the relationship is sector-dependent and the impact is time-varying. Additionally, the author showed that the effects of CDS return shocks are the most dominant during the post-Lehman crisis period. Hammoudeh and Sari [14] analyzed the relationship between the CDS, the stock market and the interest rates for banking, financial services and insurance sectors, on the example of the U.S., by using Autoregressive Distributed Lag approach (ARDL). The authors found that there is a long-run relationship between CDS, stock market and bond market, while the short-run dynamics exhibit different behaviors under the subperiods of financial distress. Fei et al. [15] examine the joint behaviors of CDS and equity markets over September 2005–March 2011, on the example of two European sectors (automotive and subordinated financial) using Markov-switching bivariate copula, and report significant negative comovements between CDS and stock index returns, which are time-varying and non-linear.

Several papers focused on the relative importance of price discovery between CDS and stock markets at the sectoral and market-wide level and the evidence are mixed. Among authors that subscribe to the conclusion that stock markets tend to lead the CDS market, we can mention Fung et al. [16], Narayan et al. [17] and Tolikas and Tapaloglou [5]. For instance, Narayan et al. [17], using a panel vector error correction model, show that the stock market contributes to price discovery in most sectors while the CDS market leads the price discovery in only a few sectors. The authors also state that the price discovery process was affected by the 2007 global financial crisis but the stock market still dominated the price discovery process. In a recent work, Tolikas and Tapaloglou [5] show that stock market reacts faster to new information, on the example of nine economic sectors and four geographical regions (North America, Europe, the UK and Asia), over the period January 2008–June 2014, based on VAR and impulse response function. Other studies ([18,19]) base their conclusions on the leading role of the CDS market with respect to the stock market.

Considering the CDS–stock connection concentrated on sovereign CDSs, a few studies have examined the relationship between CDS spreads and the stock market at the country level ([4,20,21,22,23,24]). Estimating a vector autoregressive generalized conditional heteroskedastic VAR-BEKK(Baba, Engle, Kraft and Kroner) model, with the purpose of explaining the dependency structures between credit spreads and equity returns of the European market (based on iTraxx Europe index, the Dow Jones Euro Stoxx 50 index and the Dow Jones VStoxx index), Schreiber et al. [20] found that the negative relationship between asset prices and credit spreads is valid only for the pre-crisis period. In addition, the authors show that, during the financial crisis, equity returns are not a significant predictor of spreads, while CDS lead changes in equity market volatility. Coronado et al. [21] analyzed the relationship between sovereign CDSs and stock indexes for eight European countries (France, Greece, Germany, Ireland, Italy, Portugal, Spain and the UK) during the period 2007–2010, based on panel VAR methodology. The authors found that the stock market has the leading role in the incorporation of new information, but during subsamples of financial distress, the key role was assumed by sovereign CDS markets. Corzo Santamaría et al. [22] examined the transfer of risk between the private and public sectors on the example of developed European financial markets (Spain, Portugal, Italy, France, Ireland, United Kingdom, Greece, Germany, Austrian, Belgium, Netherlands, Finland and Denmark) and found that 2008–2009 equity markets assumed the process of incorporation of new information, but during the sovereign debt crisis, this leading role was taken over by sovereign CDS markets. Ngene et al. [4] employed a threshold cointegration and threshold vector error correction model based on a sample of 13 emerging economies (namely Brazil, Bulgaria, China, Hungary, Indonesia, Malaysia, Mexico, Philippines, Poland, Russia, South Africa, Thailand and Turkey) in order to test the relationship between sovereign CDS and equity markets. The results highlight a cointegration relationship between the CDS and the equity markets. Shear et al. (2017) performed an analysis of the relationship between the CDS and the stock market on the example of an emerging economy (Pakistan), by handling outliers via the split sample skewness based boxplot technique. The authors report a negative correlation between the CDS and the equity index and, in addition, a bidirectional causality between both markets, based on the Granger causality test.

The empirical literature that dealt with the CDS–stock market connections has applied multivariate time series models, including the Vector Autoregressive (VAR) model ([5,13,20,21]), the Vector Error Correction Model ([4,17]), the Granger causality tests ([6,24]), Autoregressive Distributed Lag approach ([14]) and, recently, new econometric tools such as Markov regime-switching models ([15]), quantile-on-quantile methodology and the nonparametric causality-in-quantiles tests ([11]) and transfer entropy ([12]). The concept of entropy is used to depict nonlinear dependence within the financial returns series [25].

What we have learned so far from the literature on the proposed topic is the fact that normal and distress periods may have an important role in alternating risk transfer between the private and public sectors. It is worth mentioning that we identified plenty of room for more contribution to the CDS–stock prices relationship, at the country level in emerging Europe, especially after the occurrence of the 2008 financial and 2010 sovereign debt crises. Therefore, our contribution to the literature is timely, since most of the previous research has concentrated either on the firm or on the sectoral level CDS. Moreover, we analyze the impact of the ban on outright short selling of sovereign CDS on the CDS–stock market lead–lag relationship, comparing the findings obtained for the post-ban period with those obtained prior to the ban.

## 3. Data and Econometric Methodology

### 3.1. Data Overview and Stochastic Properties

We used daily data on stock indexes (daily closing price) and 5-year sovereign CDS spreads denominated in USD. Data was retrieved from the Eikon database for the period January 1, 2008, to April 18, 2018. Regarding the sample period, all countries had the same starting date (January 1, 2008), except the Czech Republic and Lithuania for which the data was publicly available from February 29, 2008, onwards. The beginning of the sample was driven by the availability of liquid data of the sovereign CDS for emerging European markets. Our sample contained data for nine countries from the CEE region: Croatia, Czech Republic, Hungary, Latvia, Lithuania, Poland, Romania, Slovakia and Slovenia. The data set covered 2687 daily quotes for each of the time series for Croatia, Hungary, Poland, Romania, Slovakia and Slovenia and, respectively 2644 for Czech Republic and Lithuania. As a benchmark for the stock market, daily closing prices of the country’s main stock index was employed, expressed in national currency: CROBEX (Croatia), SE PX (Czech Republic), BUX (Hungary), OMX Riga (Latvia), OMX Vilnius (Lithuania), WIG (Poland), BET (Romania), SAX (Slovakia) and SBITOP (Slovenia). In line with the academic literature in the field ([4,6,17]), we used the midpoints between the quoted bid and ask spreads for the US dollar-denominated CDS with a 5-year maturity, which represents the most traded currency in this market [22]. According to ISDA Swaps Info Quarterly Review [26], in 2017, U.S. dollar-denominated index CDS represented 63.0% of index CDS traded notional and 68.2% of trade count, while Euro-denominated transactions had a share of 36.8% and 31.4% of traded notional and trade count, respectively. Widely used in empirical analyses, 5-year credit instruments are considered to be more liquid and represent the largest proportion of the CDS market. There are a few empirical papers exploiting the lead–lag stock–CDS markets relationship, which rely on daily returns to estimate models ([5,8,10,15]).

Our sample of countries is motivated both by the need to fill in the gap in the literature regarding the lead–lag relationship between CDS and stock prices, and by the need to find useful insights when employing a set of risky countries (with a high CDS premium) and, respectively, a set of safer countries (with a low CDS premium), as a key of control. Similar as in [22], we define European countries with low spreads those countries with CDS average premium below 100 basis points (bp; i.e., the Czech Republic and Slovakia), and European countries with high spreads those with CDS average premium above 100 bp (i.e., Croatia, Hungary, Romania and Latvia).

Table 1 displays summary statistics for the CDS daily spreads in basis points. The Jacque–Berra statistics attest to rejecting the null hypothesis of a normal distribution for CDS spreads at the 1% level. The calculated averages for the CDS spreads vary across countries. The Croatian CDS spread has the largest mean value, followed by those of Hungary, Romania and Latvia, while Slovakia’s CDS has the smallest spread, reflecting lower default risk. However, Latvia and Lithuania show the largest volatility, with standard deviations of 215.70 and 149.01 bp, respectively. There was a wide dispersion within the sample of countries: the lowest CDS average spread was 8 bp for Slovenia in January-February 2008 and the highest one was 1176.30 bp for Latvia in March 2010. All CDS time series present a positively skewed distribution and exhibit leptokurtic distribution since kurtosis was positive and higher than 3.

In line with previous studies in this area [4,11,22], for the econometric analysis, we considered the logarithmic change in the CDS spreads, which “illustrates the return from speculating that the cost of default protection will change” [15]. Similarly, stock index returns are computed as the first log difference of the stock indexes [11,13]. Table 2 presents the descriptive statistics daily logarithmic changes in CDS and stock indexes.

The mean stock index returns and CDS returns were different for the analyzed countries, illustrating different profit and risk opportunities in emerging markets. The mean of stock index return series was positive for all countries, except Croatia, Czech Republic, Slovakia and Slovenia. On the other hand, Croatia shows the highest mean return (14.78%), followed by Hungary (13.18%). The Slovakian CDS yielded the highest mean returns, followed by the Slovenian CDS market. Based on skewness and kurtosis tests, all the return time series were asymmetric and fat-tailed, in line with the results reported by Ngene et al. [4] or Shahzad et al. [11].

The correlation coefficients confirmed the stylized negative association between CDS return and stock index returns, in line with previous findings in the recent literature, both on the example of emerging markets [4] and developed countries [5,15]. The correlation coefficient shows that multicollinearity does not represent a concern for the econometric approach. The weakest correlation was observed for Slovenia. We could notice a negative correlation between CDS premium and equity prices, meaning that if credit risk increased, equity prices declined. As stated by [13], CDS and stock index return shocks have different impacting abilities and these aspects were subjects of the subsequent analysis.

### 3.2. Methodology

In line with representative studies [5,9,22] the lead–lag relationship between the sovereign CDS and stock markets was analyzed based on the following bivariate Vector Autoregressive (VAR) framework:

(1) $$R_{\mathrm{CDS},t}=\alpha +\sum_{i=1}^{L}\beta_{i}R_{CDS,\;t-i}+\sum_{i=1}^{L}\gamma_{i}R_{S,\;t-i}+\varepsilon_{t}$$

(2) $$R_{S,t}=\alpha +\sum_{i=1}^{L}\beta_{i}R_{CDS,\;t-i}+\sum_{i=1}^{L}\gamma_{i}R_{S,\;t-i}+\varepsilon_{t}$$

where *R_CDS,t_* is the daily sovereign CDS spread return at time *t*, *R_S,t_* is the daily stock index return at time t and L is the lag length. The *α* is the intercept term, *β_i_* and *γ_i_* are the cross-market coefficients to be estimated respectively and ε_t_ is the error term that follows a multivariate normal distribution. The lagged terms reduce the risk of getting a spurious nexus due to “stale quotes or infrequent updates of prices” [5]. The optimal length of the lags has been determined based on the Bayesian information criterion (SIC). For diagnostic checking of estimated VAR models, tests for residual autocorrelation and heteroskedasticity have been performed. Portmanteau test for residual autocorrelation checks the null hypothesis *H_0_*: E(*u_t_,u’_t-i_*)=0, for *i* = 1, …, *h* > *L* against the alternative that at least one autocovariance and, hence, one autocorrelation is nonzero. The Breusch–Godfrey Lagrange multiplier (LM) test for h-th order residual autocorrelation assumes a model: $\;u_{t}=B_{1}^{*}u_{t-1\;}+\ldots +B_{h}^{*}u_{t-h}+{\mathrm{error}}_{t}\;$ and checks *H_0_:* $B_{1}^{*}$=...=$B_{h}^{*}=0$ vs. H_1:_ $B_{1}^{*}\neq 0\;or\ldots .B_{h}^{*}\neq 0$. The white heteroskedasticity test was employed to test the null hypothesis of no heteroskedasticity against the alternative.

The daily variables, sovereign CDS spread return (*R_CDS,t_*) and stock index return (*R_S,t_*) need to be stationary. We conducted several tests to check the stationarity of the time series: the augmented Dickey–Fuller and, respectively, breakpoint unit root test (not reported here, but available upon request). The results strongly suggest that all the return series are stationary processes at the conventional levels. The financial time series, therefore, satisfy also the conditions for transfer entropy, a non-linear causality method with required stationarity of the return series. We also employed the Johansen’s cointegration test to analyze for cointegration between the above two variables. The findings highlight that there is no long-run equilibrium between the two markets for the sample period and explain the use of the VAR system instead of a vector error correction model (VECM). The lack of cointegration means that the difference between the CDS spread return and stock index return is not zero and systematically shows persistence during the financial distress period, therefore investment strategies should be revised [27].

In line with Tolikas and Tapaloglou [5], the null hypothesis means that default risk is priced efficiently by the two markets, simultaneously. Therefore, the estimated cross-market coefficients of the lagged terms should be zero. We employed the Granger Causality and, in order to test the null hypothesis that the CDS spread returns do not lead the stock returns, we analyzed the F-statistic of the hypothesis H_0_= [β_i_]=0 for all i. In reverse, in order to test the null hypothesis that the stock returns do not lead the CDS spread return, the F-statistic of the hypothesis *H_0_= [γ_i_]=*0 for all i was examined.

Further, the accumulated impulse responses of the CDS spread return and stock index return to one standard deviation shock to each other and their lagged values were calculated. The impulse response analysis illustrates the dynamic characteristics of the lead–lag relationship between the two markets. Daily sovereign CDS spread return (R_CDS_) and daily stock index return (R_S_) were treated as endogenous variables.

According to the recent literature, the following hypotheses for the dynamic relationships between CDS spreads and stock market returns were tested:

**Hypothesis** **1.** *Sovereign CDS spreads and stock indices are interrelated and move in the opposite direction. Considering the country’s economic barometer, a volatile stock market transmits negative signals to investors regarding the economic fundamentals of that country [4]. On the other hand, the sovereign CDS spread represents an indicator of financial health, guided by a country’s economic fundamentals. Therefore, we expected sovereign CDS spreads to interrelate with the stock market. Moreover, it is demonstrated that an increase (decrease) in the stock prices is accompanied by a fall (rise) in the CDS premium [4]. We assumed the statement to be valid for the relationship between stock returns and CDS spread changes.*

**Hypothesis** **2.** *The stock market leads the price discovery process. The stock market is characterized by higher trading volume and liquidity and thus we expected to play a significant role in the price discovery process. We stated this hypothesis in line with studies by Tolikas and Tapaloglou [5], respectively Coronado et al. [21].*

**Hypothesis** **3.** *The lead–lag relationship between the sovereign CDS and stock indices is subject to change over time. Studies by Shahzad et al. [6] and Coronado et al. [21] show that the relationship between the CDS spreads and stock markets is time-varying. We assumed that it is also possible to identify a change in the price discovery dominance and, therefore, we analyzed the dynamic relationships between CDS spreads and stock market returns under abnormal market conditions (financial crisis) and, respectively market interventions (ban on outright short-selling of sovereign CDS contracts).*

## 4. Empirical Results

The lead–lag analysis between the sovereign CDS and the stock indices for CEE countries, as well as the results of the Granger causality test, are displayed in Table 3. The adequacy of the standard diagnostic tests for the estimated VAR models is reported in Appendix A Table A1 and Appendix B Table A2. According to Portmanteau, LM and white tests, the residuals did not exhibit autocorrelation and conditional heteroskedasticity. Moreover, all the estimated VAR models satisfied the stability condition, since all the roots of the characteristic polynomial were below 1. According to the empirical results reported in Table 3, a significant negative comovement between CDS and stock index returns could be observed, according to the predictions of the theoretical credit risk model, proposed by [28]. This can be explained by the fact that when a country faces increased default risk, the demand for protection increases and CDS spread rises, while the stock market is negatively impacted due to high-risk premium demand of investors [4]. This finding confirms the first hypothesis. The empirical results reveal that the lagged stock index returns have a more predictive power for the sovereign CDS spread returns for all countries, except Slovakia. Country by country analysis shows that there were many statistically significant coefficients for the lagged stock index returns that helped to explain the CDS spread returns. For example, in the case of the Czech Republic, the coefficients of the first, third and fourth lagged stock index returns had the followings values: −0.42, −0.22 and −0.26, and were statistically significant according to the t-statistics of −7.33, −3.89 and −4.41, respectively. On the other hand, the lagged CDS returns led the stock index returns in Slovakia, as shown by estimated coefficients, but the magnitude was smaller. However, in Slovakia, the lead–lag relationships became less intense and we did not find much evidence of Granger causality. Only the coefficient of the lagged two of CDS spread return is statistically significant at the 1% level with an estimated value of −0.02.

As shown by the Granger test, the null hypothesis that CDS spread is not influenced by lagged stock returns was rejected in all countries, except for Slovakia, where the Granger F-statistic registered the value of 0.11 with a *p*-value of 0.98. On the other hand, the null hypothesis that stock returns are not influenced by lagged CDS spread returns was rejected in all countries, except Latvia and Romania. Therefore, we found evidence of the presence of bidirectional causality between sovereign CDS and stock markets in Croatia, Czech Republic, Hungary, Lithuania, Poland and Slovenia. Overall, our results provided evidence that the stock market in CEE countries was more efficient in pricing the changes in default risk compared to the CDS market, which shows that stock prices dominated CDS spread.

**Figure 1:**
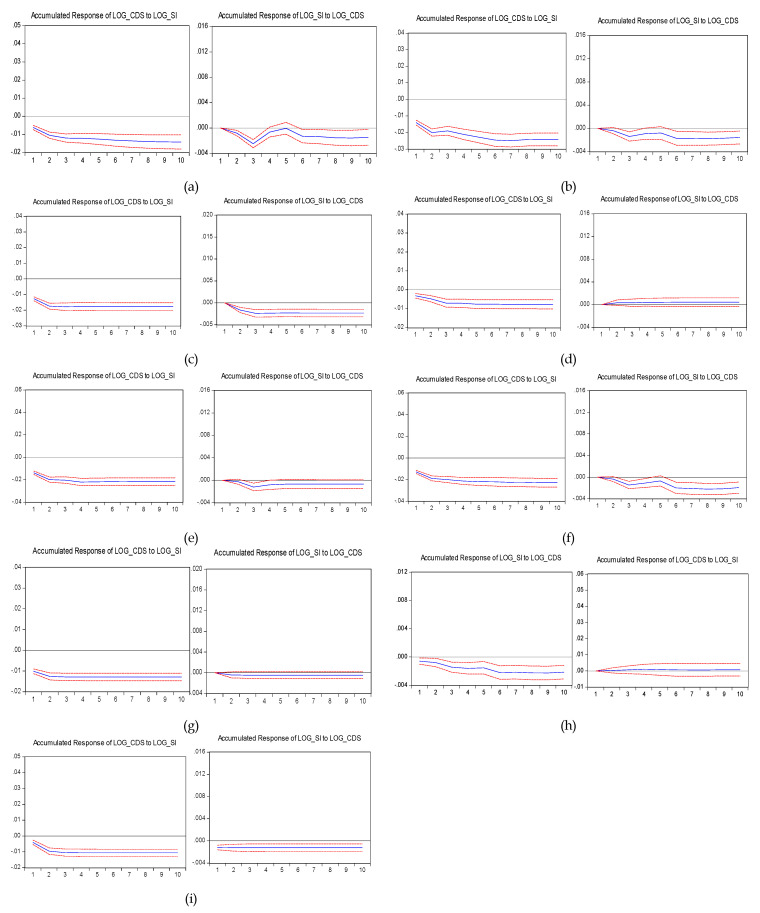
Impulse responses to one standard deviation for up to 10 lags over the period January 1, 2008–April 18, 2018 (%). Note: Countries: (**a**) Croatia; (**b**) Czech Republic; (**c**) Hungary; (**d**) Latvia; (**e**) Lithuania; (**f**) Poland; (**g**) Romania; (**h**) Slovakia and (**i**) Slovenia. The blue line displays the accumulated response of the CDS spread to one standard deviation shock in the return of the stock market and respectively, the accumulated response of the stock market return to one standard deviation shock in the CDS spread. The red short-dashed lines are the 95% confidence intervals. In the case of Latvia and Romania, stocks’ response to CDS was not statistically significant. CDSs’ response to stocks was not statistically significant in the case of Slovakia.

In order to capture the dynamic properties of the relationship between the stock and CDS markets, we computed the accumulated impulse responses of the sovereign CDS and stock market returns. The shock amounts to one standard deviation of the past values. The results of the accumulated impulse responses between the CDS spread returns and stock market returned up to 10 lags are shown in Figure 1. The analysis highlighted that in all countries the accumulated responses of the CDS spread to a one standard deviation shock in the returns of the stock market show higher values compared to those of the stock market reaction to a shock in the returns of the CDS spread. For instance, the responses of the CDS spread for Croatia to a one standard deviation shock in the return of the stock index was 1.42% compared to 0.15% for the stock index counterpart. These findings were in line with the Granger test results, indicating that the influence of the stock index on CDS spread was more pronounced than the other way around. The results indicate that the stock market outputs more information compared to the CDS market. 

**Figure 2:**
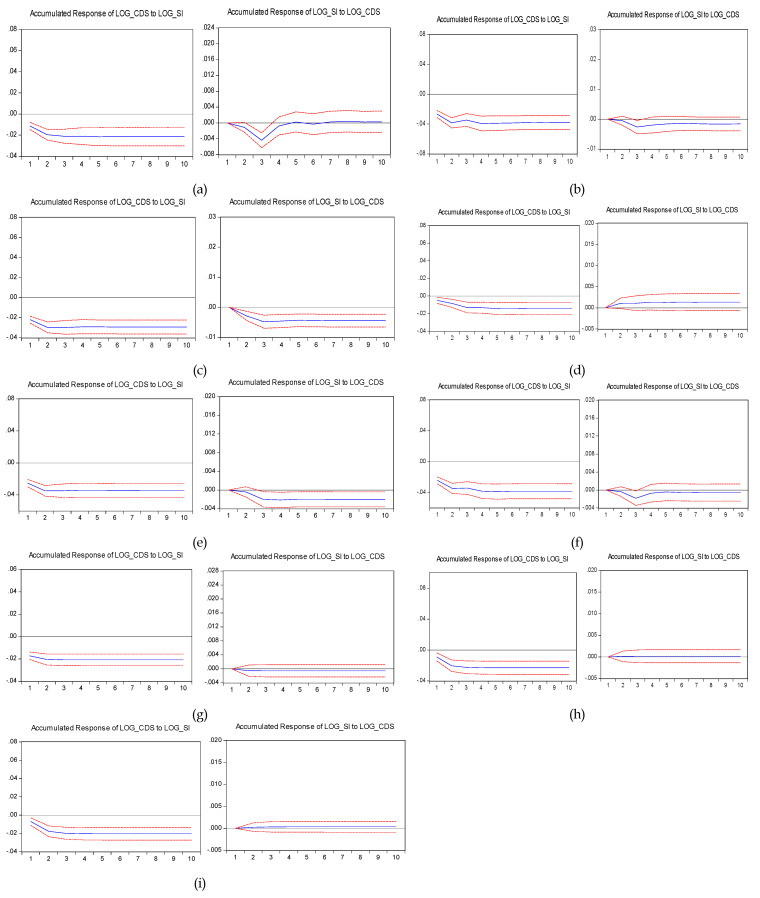
Impulse responses to one standard deviation for up to 10 lags over the period January 1, 2008–March 03, 2011 (%). Note: Countries: (**a**) Croatia; (**b**) Czech Republic; (**c**) Hungary; (**d**) Latvia; I Lithuania; (**f**) Poland; (**g**) Romania; (**h**) Slovakia; and(**i**) Slovenia. The blue line displays the accumulated response of the CDS spread to one standard deviation shock in the return of the stock market and respectively, the accumulated response of the stock market return to one standard deviation shock in the CDS spread. The red short-dashed lines are the 95% confidence intervals. In the case of Latvia, Romania, Slovenia and Slovakia, the stocks’ response to CDS was not statistically significant.

**Figure 3:**
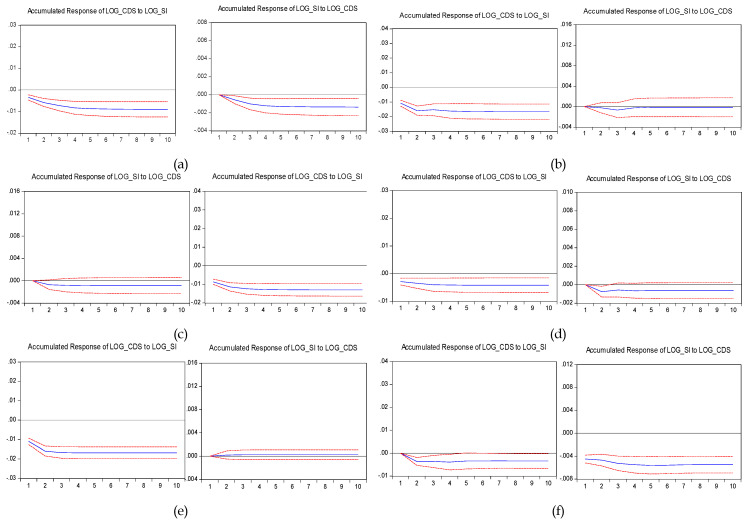

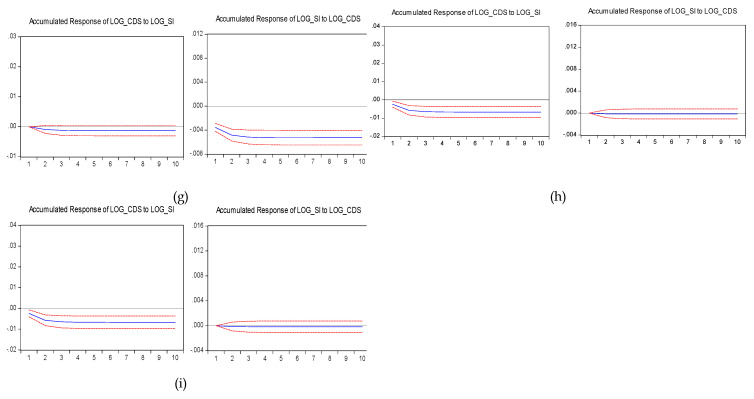
Impulse responses to a one standard deviation shock for up to 10 lags over the period April 1, 2011–June 30, 2014 (%). Note: Countries: (**a**) Croatia; (**b**) Czech Republic; (**c**) Hungary; (**d**) Latvia; (**e**) Lithuania; (**f**) Poland; (**g**) Romania; (**h**) Slovakia and (**i**) Slovenia. The blue line displays the accumulated response of the CDS spread to one standard deviation shock in the return of the stock market and respectively, the accumulated response of the stock market return to one standard deviation shock in the CDS spread. The red short-dashed lines are the 95% confidence intervals. In the case of the Czech Republic, Latvia, Lithuania, Slovenia and Slovakia, the stocks’ response to CDS was not statistically significant. The CDSs’ response to stocks was not statistically significant in the case of Hungary.

We also reproduced our methodology and results under financial distress conditions. Therefore, we split our sample period into two subperiods, as follows: January 01, 2008, to March 03, 2011, which corresponds to the global financial crisis (GFC) and respectively, April 01, 2011, to June 30, 2014, which corresponds to sovereign debt crisis (SDC). The results of the two subperiods are reported in Table 4, Table 5, Table 6 and Table 7 and, respectively Figure 2 and Figure 3. The results for the GFC period support the previous findings. For example, in Croatia, the first two coefficients for lagged stock returns were statistically significant and took the values of −0.36 and −0.06 and t-statistics of −3.91 and −2.67, respectively. Moreover, according to the F-statistic, which took the value of 5.29 with a *p*-value of 0.00, the hypothesis that stock returns do not influence the CDS spreads was rejected. The analysis over the first subperiod suggests that in 7/9 countries the results were consistent with a leading role of the stock market. Only in Romania and Slovakia, there was no significant information flow between the markets. There was reciprocal Granger causality for five out of nine countries, and we could talk about a feedback process in Croatia, the Czech Republic, Hungary, Lithuania and Poland. In all of the countries, except Romania and Slovakia, the null hypothesis that the lagged stock returns do not influence the CDS spreads was rejected. On the other side, for the influence of the lagged CDS spreads, this was only applicable in Croatia, the Czech Republic, Hungary, Lithuania and Poland, where the F-statistic of the Granger test took *p*-values ranging from 0.00 to 0.02.

When analyzing the second subperiod (April 1, 2011–June 30, 2014) we appreciated a change in the lead–lag relationship. The results are reported in Table 5 and, respectively Figure 3. During the sovereign debt crisis, the leading role of the stocks became weaker, the reciprocal Granger causality being reported only in the case of Croatia and Poland, as shown in Table 5. The CDS market took the lead in Romania and Slovenia, as the first coefficients of the lagged CDS spread returns were statistically significant and took the values of −0.05 and −0.10, respectively. The impulse response analysis reported in Figure 3 highlights that in Romania and Slovenia, the accumulated responses of the stock index to a one standard deviation shock in the returns of CDS spread show higher values compared to those of the CDS market reaction to a one standard deviation shock in the returns of the stock index. Despite a few pieces of evidence of bidirectional feedbacks between CDS and stock returns, the unidirectional information flow from stock to CDS returns could be noticed also for the Czech Republic and Latvia. This supports more evidence in the favor of Granger causality from stock returns towards CDS returns compared to the opposite relationship. It can be observed that, during the sovereign debt crisis, the price discovery process tended to migrate between CDS and stock markets. During periods of financial distress, the demand for credit protection was high and, therefore, the liquidity moved towards the CDS market. 

Similar to the findings reported by previous studies [5,21], our results highlighted the leading capacity of the stock market over the CDS market and showed the ability of the stock market to incorporate faster and efficiently new information. A possible explanation could be the fact that the liquidity of the stock market was higher compared to the sovereign CDS market. On the other hand, in emerging markets, the CDS investor base is dominated by banks [29]. The banking system provides loans to public and private sectors and, therefore, buys protection—CDS to hedge the exposures, but do not trade the CDS. As a consequence, the stock market is characterized by a higher trading volume and liquidity and plays a significant role in the price discovery process.

The sovereign CDS market has undergone a change during the analyzed period, respectively the policy-makers introduced EU regulation 236/2012, the permanent ban on naked sovereign CDS contracts. It is interesting to see if the adoption of the ban on naked CDS trading influences the leadership role or entropy transfer between the markets. Therefore, we investigated the effect of the ban on the lead–lag relationship between sovereign CDS and stock indices. In order to perform the analysis, in line with [7], we split our data into a pre-ban period spanning from January 2008 to 13 March 2012, and respectively, a post-ban period from 14 March 2012 to 18 April 2018. The results are displayed in Table 6 and Table 7. The findings provided evidence for the leading role of the stock market in the pre–ban period for all countries, except Romania and Slovakia. Additionally, the results in Table 6 confirmed significant information flow in both ways for five out of nine countries (Croatia, Czech Republic, Hungary, Lithuania and Poland).

We re-estimated the models for data under the post–ban period and the results reported in Table 7 clearly indicate that the causal linkages between CDS and stock markets were less evident compared to the pre–ban period. Following the adoption of the naked CDS ban, we found the one-way information flow from stock to CDS returns for Croatia, Czech Republic, Hungary, Poland, Romania and Slovenia. The results for the post–ban period highlighted that, as for the pre–ban period, the pricing leadership of the stock market in most of the countries. Further, there are notably few evidence of a feedback effect from the CDS market to the stock market. A possible explanation could be the fact that short-selling bans had a negative impact on liquidity, as shown by [30,31].

### Robustness Test Using Alternative Measures

Sovereign CDS contracts are expressed in another currency from one of the deliverable obligations [7], therefore CDS on the euro area is highly concentrated in US dollars denominated segment. The empirical research employs the most liquid CDS segment, expressed in USD. However, we analyzed the evolution of dollar- and euro-denominated CDS contracts for the entire period and we found a similar behavior, with the mention that the premium of euro-denominated sovereign CDS was cheaper. In order to check the robustness of the results, we retook the analysis over the period October 8, 2008–April 18, 2018, using 5-year EUR denominated CDS for Slovakia and Slovenia, since these countries are Eurozone members, and the comparison lets us check whether we got a similar output. The empirical findings reported in Table 8 were broadly in line with the benchmark results, as we did not find evidence of Granger causality between sovereign CDS and stock markets in Slovakia, while, in Slovenia, the stock market and the CDS market incorporated the default risk.

Tolikas and Tapaloglou [5] identified several reasons to explain the dominant role of the stock market in pricing new information: increased trading costs in the CDS market, the slow dissemination of information across economic sectors, the possible existence of nonsynchronous trading and a nonlinear nexus between the CDS and the stock market.

## 5. Conclusions

This study examined the lead–lag relationship between sovereign CDS and stock markets in nine emerging economies from the CEE region, namely Croatia, Czech Republic, Hungary, Latvia, Lithuania, Poland, Romania, Slovakia and Slovenia, over the period January 2008–April 2018. Based on daily data on stock indexes (daily closing price) and 5-year sovereign CDS spreads denominated in USD, the analysis deploys a Vector Autoregressive model, focusing on the intermarket causal linkage between the credit and stock markets. The empirical results revealed the presence of bidirectional feedback between sovereign CDS and stock markets in Croatia, Czech Republic, Hungary, Lithuania, Poland and Slovenia. The two-way feedback supposes that information on one market can be used to predict the movement of the other. Overall, our findings provided evidence that the stock markets in CEE countries incorporated faster the modifications in default risk compared to the CDS markets and therefore led the price discovery. Therefore, the information was reflected first in the stock market, meaning that investors managed their portfolios in line with stock markets’ evolution. The results suggest a transfer entropy of risk from the private to the public sector over the period 2008–2018.

We focused on the price discovery, meaning the pricing of new information at the right time, under abnormal market conditions (financial crisis) and, respectively market interventions (ban on outright short-selling of sovereign CDS contracts). During the Global Financial Crisis, for all the countries analyzed except Romania and Slovakia, results were consistent with the leading role of the stock market. In line with the previous studies [22], our results show that, during the latest global financial crisis, the stock markets in the emerging economies led the process of incorporation of new information. However, during the European Sovereign Debt Crisis, we appreciated a change in the lead–lag relationship. The leading role of stocks became weaker, but still more evidence was found in favor of Granger causality from stock returns towards CDS returns compared to the opposite relationship. For two countries in the sample (Romania and Slovenia), the results suggest a transfer entropy of risk from the public to the private sector during the sovereign debt crisis. A possible explanation for these fluctuations would be liquidity migration between CDS and stock markets.

Moreover, we investigated the impact of the bans on price discovery, comparing the results obtained for the post–ban period with those to the ban introduction. The findings provide evidence for the leading role of the stock market in the pre–ban period for all countries in our sample, except for Romania and Slovakia, while the post–ban period the causal linkages between CDS and stock markets were less evident. Under the post-ban period, there was notably little evidence of a feedback effect from the CDS market to the stock market. This finding supposed that the stock market was more efficient in the pricing default risk compared to the CDS market. Therefore, it means that there were internal information asymmetries between market players since, in emerging markets, the major banks dominated the sovereign CDS market.

The results are useful for policymakers, traders and long-term investors, who need to act under different market conditions. Policymakers may be interested in the subject because the financial crisis highlighted that speculation in sovereign markets might spread risks to stock markets. Credit risk information tends to alternate across markets, and therefore, policymakers should formulate sound and synchronized regulatory policies. Therefore, insights on the CDS–stock market lead–lag relationship provide information about the dependence structure of the two markets, which are useful for better supervision of risk transfer and for implementing policies towards pricing efficiency. Market participants should seek information in both markets while engaging in hedging or trading, with a preponderant view on the stock market [11]. The results are in line with market selection theory, which states that informed traders choose only one market to trade [32], because of several reasons: costs, liquidity, knowledge about legislation [5] and, therefore, the favored market incorporates the new information. The findings highlighted that, in the CEE countries, the informed traders of default risk preferred the stock market, while uninformed traders activated, mostly, in the CDS market. Since the CDS market is influenced by large investors (i.e., banks and insurance companies), they may have informational advantages derived from trading in a less transparent market. Therefore, the CDS market is characterized by a lack of informed traders, while the stock market represents the preferred channel of informed trading. Additionally, the CDS traders can look at the evolution of the stock market, if there are concerns about pricing the default risk. Therefore, the existence of the lead–lag relationship between the two markets highlights informational asymmetries and traders can identify profitable opportunities. Sovereign CDS can be used to hedge government bond position, speculation or naked position on the corresponding market. The results present relevant practical implications for the asset management industry, in terms of managing national credit risk exposure and rebalancing investments decisions. The current research highlights that informed investors respond quickly to new information in the stock market, therefore, they are able to better evaluate the probabilities of default and the outcome is an improved risk-return structure of portfolios. On the other hand, asset managers of banks use CDS for market-making activities and for managing exposures to sovereigns. Additionally, asset managers of hedge funds and pension funds can use CDS in order to offer protection against exposures to the sovereign bond market. Accordingly, asset managers should pay attention to risk comovements between markets in the process of financial portfolios diversification [33].

Additionally, investors may be interested to know which market is able to incorporate news first. Understanding the dynamics of market interplay under different market conditions (financial crisis) or market interventions (ban on naked CDS) offers to investors the possibility to receive signals about the sovereign credit risk reversals. The ups and downs of stock markets can be used as an indicator for forecasting the development of the sovereign CDS market, therefore our study contributes to the informational background of investors in choosing the investment strategies. The analysis is also useful for economic and financial stability.

The findings of the current empirical research are dependent on the 5-year maturity of the credit derivatives, while the investors are trading all types of maturities. An examination of the other maturities in the lead–lag nexus between the CDS and the stock market could offer interesting insights.

## Figures and Tables

**Table 1:** Descriptive statistics for the daily sovereign Credit Default Swap (CDS) spreads.

	N	Mean	Max	Min	SD	Skew	Kurt	J–B Stats
Croatia	2687	268.06	592.50	68.50	109.51	0.54	3.39	146.52 ***
Czech Republic	2644	75.33	350.00	33.00	45.73	2.23	9.79	7286.52 ***
Hungary	2687	250.72	729.89	55.00	138.94	0.95	3.11	406.36 ***
Latvia	2687	224.48	1176.30	41.85	215.70	1.86	6.05	2585.95 ***
Lithuania	2644	186.25	850.00	46.09	149.01	1.80	6.62	2879.17 ***
Poland	2687	113.00	421.00	26.00	67.31	1.53	5.10	1538.53 ***
Romania	2687	226.34	767.70	84.17	133.31	1.48	5.28	1566.99 ***
Slovakia	2687	85.89	306.01	13.00	64.26	1.75	5.43	2039.63 ***
Slovenia	2687	148.02	488.58	8.00	108.51	1.21	3.39	667.51 ***

Note: N, max., min., SD, skew, kurt and J–B stats stand for the number of observations, maximum, minimum, standard deviation, skewness, kurtosis, and the Jarque–Bera normality test, respectively. *** indicates that the null hypothesis of normality is rejected at the 1% significance level.

**Table 2:** Descriptive statistics for daily logarithmic changes in sovereign CDS and stock index returns.

	Stocks						CDS						
	Mean (%)	Max (%)	Min (%)	SD (%)	Skew	Kurt	Mean (%)	Max (%)	Min (%)	SD (%)	Skew	Kurt	ρstocks, CDS
Croatia	−0.04	14.78	−10.76	1.17	−0.01	25.43	0.01	87.50	−25.05	3.08	8.49	2.54	−0.18(0.00)
Czech Republic	−0.01	12.36	−16.19	1.42	−0.63	21.41	−0.01	68.94	−50.19	4.03	2.42	89.85	−0.28(0.00)
Hungary	0.01	13.18	−12.65	1.56	−0.08	11.69	0.02	58.92	−25.57	3.34	3.66	64.63	−0.34(0.00)
Latvia	0.02	11.60	−7.86	1.27	0.66	13.56	−0.04	92.28	−34.84	3.20	8.78	278.40	−0.07(0.00)
Lithuania	0.01	11.00	−11.94	1.06	−0.40	30.13	−0.04	78.79	−38.53	3.05	7.13	206.51	−0.16(0.00)
Poland	0.00	6.08	−8.29	1.20	−0.50	7.87	0.03	105.40	−65.39	4.23	6.01	188.37	−0.34(0.00)
Romania	0.00	10.56	−13.12	1.50	−0.74	14.75	0.00	53.81	−36.35	3.06	2.95	74.29	−0.28(0.00)
Slovakia	−0.01	11.88	−14.81	1.17	−1.18	26.33	0.05	133.12	−44.63	4.19	12.23	4.02	−0.06(0.00)
Slovenia	−0.04	8.36	−8.43	1.09	−0.54	12.04	0.04	55.96	−36.10	3.68	1.88	50.63	−0.05(0.00)

Note: The ‘Mean’, ‘Min’, and ‘Max’, ‘SD’, ‘Skew’ and ‘Kurt’ are the daily average, minimum, maximum, standard deviation, skewness and kurtosis values, respectively, based on the returns of CDS and equity (log(Pt /Pt-1) for the period January 1, 2008–April 18, 2018. ρstocks, CDS represents the Spearman rank correlation coefficient between the returns of CDS and equity (the *p*-value of the null hypothesis that the estimated correlation coefficient is statistically equal to zero provided in parentheses).

**Table 3:** The lead–lag relationship between sovereign CDS and stock indices for Central and Eastern Europe (CEE) countries over the period January 1, 2008–April 18, 2018.

	Lagged CDS Spread					Lagged Stock Returns					Granger
	$\beta_{1}$	$\beta_{2}$	$\beta_{3}$	$\beta_{4}$	$\beta_{5}$	$\gamma_{1}$	$\gamma_{2}$	$\gamma_{3}$	$\gamma_{4}$	$\gamma_{5}$	
**Croatia**											
CDS	0.13***	0.06***	−0.02	−0.07***	0.09***	−0.31***	−0.02	0.00	−0.05	−0.00	8.00 (a)
	(6.66)	(3.22)	(−1.16)	(−3.62)	(4.70)	(−5.96)	(−0.39)	(0.15)	(−1.07)	(−0.06)	(0.00)
Stock	−0.02***	−0.05***	−0.07***	0.00	−0.04***	0.08***	−0.09***	0.11**	0.04**	0.02	36.00 (b)
	(−3.76)	(−6.96)	(−10.03)	(1.07)	(−5.42)	(4.21)	(−4.64)	(5.86)	(2.49)	(1.06)	(0.00)
**Czech Republic**											
CDS	0.00	−0.00	−0.03	0.11***	0.05***	−0.42***	0.10	−0.22***	−0.26***	−0.10	17.39 (a)
	(0.06)	(−0.27)	(−1.87)	(5.78)	(2.73)	(−7.33)	(1.77)	(−3.89)	(−4.41)	(−1.71)	(0.00)
Stock	−0.01	−0.02***	0.01	−0.00	−0.02	0.06***	−0.10***	0.01**	−0.00	−0.01	6.20 (b)
	(−1.52)	(−3.58)	(1.88)	(−0.14)	(−3.67)	(3.19)	(−4.84)	(−1.81)	(0.45)	(−0.13)	(0.00)
**Hungary**											
CDS	0.11***	0.02	-	-	-	−0.21***	0.03	-	-	-	12.09 (a)
	(5.35)	(0.96)				(−4.81)	(0.82)				(0.00)
Stock	−0.05***	−0.01**	-	-	-	−0.00	−0.12***	-	-	-	18.20 (b)
	(−5.51)	(−2.03)				(−0.28)	(−5.73)				(0.00)
**Latvia**											
CDS	−0.08***	0.20***	-	-	-	−0.14***	−0.15***	-	-	-	8.98 (a)
	(−4.36)	(10.65)				(−3.06)	(−3.13)				(0.00)
Stock	0.01	0.00	-	-	-	−0.06***	0.03	-	-	-	1.02 (b)
	(1.43)	(0.19)				(−3.25)	(1.43)				(0.35)
**Lithuania**											
CDS	−0.03	0.10***	0.01	−0.05***	0.15***	−0.19***	−0.15***	−0.28***	0.14	0.16	12.39 (a)
	(−1.70)	(5.24)	(0.33)	(−2.87)	(7.87)	(−3.37)	(−2.74)	(−4.97)	(0.52)	(0.85)	(0.00)
Stock	−0.04***	−0.01	−0.02***	−0.01	−0.03***	0.15***	0.03	0.06***	0.04**	−0.06***	16.14 (b)
	(−6.05)	(−1.65)	(−3.61)	(−0.75)	(−4.63)	(7.47)	(1.39)	(3.03)	(2.11)	(−3.07)	(0.00)
**Poland**											
CDS	0.02	0.02	−0.07	−0.15	0.16	−0.47***	−0.01	−0.21***	−0.22***	0.09	13.28 (a)
	(1.28)	(0.96)	(−4.01)	(−7.58)	(8.13)	(−6.88)	(−0.15)	(−2.99)	(−3.21)	(1.38)	(0.00)
Stock	−0.01	−0.02***	−0.01**	0.01	−0.03***	0.09	−0.09	0.01	0.01	−0.04	13.19 (b)
	(−1.65)	(−4.63)	(−2.05)	(1.23)	(−6.43)	(4.64)	(−4.58)	(0.42)	(0.93)	(−2.33)	(0.00)
**Romania**											
CDS	0.05	-	-	-	-	−0.12***	-	-	-	-	9.69 (a)
	(2.64)					(−3.11)					(0.00)
Stock	−0.01	-	-	-	-	0.04	-	-	-	-	2.26 (b)
	(−1.50)					(2.09)					(0.13)
**Slovakia**											
CDS	0.17***	0.03	−0.03	−0.14***	0.17***	0.02	0.03	0.03	−0.01	−0.01	0.11 (a)
	(9.20)	(1.84)	(−1.48)	(−7.77)	(9.34)	(0.39)	(0.42)	(0.44)	(−0.23)	(−0.17)	(0.98)
Stock	−0.01	−0.01***	−0.00	0.00	−0.01***	−0.11***	−0.02	−0.04***	−0.03	−0.01	4.61 (b)
	(−1.19)	(−2.89)	(−0.66)	(0.34)	(−3.45)	(−5.83)	(−1.04)	(−2.51)	(−1.63)	(−0.68)	(0.00)
**Slovenia**											
CDS	0.03	-	-	-	-	−0.26***	-	-	-	-	21.63 (a)
	(1.67)					(−4.65)					(0.00)
Stock	−0.01***	-	-	-	-	−0.19***	-	-	-	-	6.77 (b)
	(−2.60)					(−10.24)					(0.00)

Note: The t-statistics are provided in parentheses. ** *p* < 0.05; *** *p* < 0.01. Granger is the F-statistic (*p*-value in parentheses) of the null hypothesis that all estimated coefficients are statistically equal to zero. (a): Row means that the stock market does not Granger cause the CDS market; (b): Row means that the CDS market does not a Granger cause the stock market.

**Table 4:** The lead–lag relationship between sovereign CDS and stock indices for CEE countries over the period January 1, 2008–March 03, 2011.

	Lagged CDS Spread					Lagged Stock Returns					Granger
	$\beta_{1}$	$\beta_{2}$	$\beta_{3}$	$\beta_{4}$	$\beta_{5}$	$\gamma_{1}$	$\gamma_{2}$	$\gamma_{3}$	$\gamma_{4}$	$\gamma_{5}$	
**Croatia**											
CDS	0.14***	0.05	−0.03	-	-	−0.36***	−0.06***	−0.01	-	-	5.42 (a)
	(4.06)	(1.39)	(−1.04)			(−3.91)	(−2.67)	(−0.16)			(0.00)
Stock	−0.02	−0.06***	0.09	-	-	0.10***	−0.1324***	0.16***	-	-	23.64 (b)
	(−1.81)	(−4.76)	(0.98)			(3.05)	(−3.83)	(4.85)			(0.00)
**Czech Republic**											
CDS	0	0	−0.06	-	-	−0.57***	0.22	−0.37***	-	-	10.20 (a)
	(−0.23)	(−0.08)	(−1.60)			(−4.59)	(1.80)	(−2.97)			(0.00)
Stock	0	−0.03***	0.01	-	-	0.08**	−0.14***	−0.05	-	-	3.19 (b)
	(−0.67)	(−2.78)	(1.01)			(2.07)	(−3.61)	(−1.33)			(0.02)
**Hungary**											
CDS	0.10***	0.02	-	-	-	−0.24***	0.07	-	-	-	4.26 (a)
	(2.79)	(0.58)				(−2.71)	(0.83)				(0.01)
Stock	−0.06***	−0.03***	-	-	-	0	−0.18***	-	-	-	10.42 (b)
	(−3.82)	(−2.17)				(−0.05)	(−4.97)				(0.00)
**Latvia**											
CDS	−0.11***	0.23***	-	-	-	−0.22***	−0.21***	-	-	-	5.17 (a)
	(−3.38)	(6.99)				(−2.36)	(−2.27)				(0.00)
Stock	0.02	0	-	-	-	−0.02	0	-	-	-	1.54 (b)
	(1.71)	(0.29)				(−0.83)	(0.03)				(0.21)
**Lithuania**											
CDS	−0.10***	0.10***	0.02	-	-	−0.30***	−0.1	−0.46***	-	-	9.02 (a)
	(−2.79)	(2.91)	(0.54)			(−2.59)	(−0.89)	(−3.88)			(0.00)
Stock	−0.05***	−0.01	−0.03***	-	-	0.23***	0	0.07***	-	-	11.78 (b)
	(−4.99)	(−1.1831)	(−2.60)			(6.20)	(0.1874)	(2.11)			(0.00)
**Poland**											
CDS	0	0	−0.09***	-	-	−0.66***	0.11	−0.46***	-	-	8.161 (a)
	(0.12)	(0.17)	(−2.63)			(−4.19)	(0.75)	(−2.9)			(0.00)
Stock	0	−0.02***	0.01**	−	-	0.10***	−0.12***	0.08**	-	-	4.29 (b)
	(−0.6882)	(−2.63)	(2.28)			(2.87)	(−3.24)	(2.30)			(0.00)
**Romania**											
CDS	0.03	-	-	-	-	−0.11	-	-	-	-	2.17 (a)
	(1.05)					(−1.45)					(0.14)
Stock	−0.01	-	-	-	-	0.03	-	-	-	-	0.48 (b)
	(−0.65)					(0.90)					(0.48)
**Slovakia**											
CDS	0.17***	0.02	−0.05	-	-	0.04	0.13	0.02	-	-	0.23 (a)
	(4.99)	(0.65)	(−1.61)			(0.24)	(0.79)	(0.13)			(0.87)
Stock	0	−0.01**	0	-	-	−0.02	0.03	−0.02	-	-	2.12 (b)
	(−0.09)	(−2.31)	(−0.26)			(−0.73)	(0.96)	(−0.75)			(0.09)
**Slovenia**											
CDS	0	-	-	-	-	−0.80***	-	-	-	-	19.14 (a)
	(0.15)					(−4.3)					(0.00)
Stock	0	-	-	-	-	0.18***	-	-	-	-	0.053 (b)
	(0.25)					(5.32)					(0.81)

Note: The t-statistics are provided in parentheses. ** *p* < 0.05; *** *p* < 0.01. Granger is the F-statistic (*p*-value in parentheses) of the null hypothesis that all estimated coefficients are statistically equal to zero. (a) Row means that the stock market does not Granger cause the CDS market; (b) Row means that the CDS market does not Granger cause the stock market.

**Table 5:** The lead–lag relationship between sovereign CDS and stock indices for CEE countries over the period April 1, 2011–June 30, 2014.

	Lagged CDS Spread					Lagged Stock Returns					Granger
Z	$\beta_{1}$	$\beta_{2}$	$\beta_{3}$	$\beta_{4}$	$\beta_{5}$	$\gamma_{1}$	$\gamma_{2}$	$\gamma_{3}$	$\gamma_{4}$	$\gamma_{5}$	
**Croatia**											
CDS	0.14***	0.13***	−0.01	-	-	−0.28***	−0.06	−0.08	-	-	4.00 (a)
	(4.34)	(4.01)	(−0.14)			(−3.03)	(−0.72)	(−0.87)			(0.00)
Stock	−0.03**	−0.01	0	-	-	0.06	−0.03	−0.03	-	-	5.76 (b)
	(−2.53)	(−1.60)	(−0.26)			(1.72)	(−1.08)	(−0.97)			(0.00)
**Czech Republic**											
CDS	0.05	0.04	0.07**	-	-	−0.32***	0.13	−0.03	-	-	5.95 (a)
	(1.54)	(1.15)	(2.05)			(−4.27)	(1.77)	(−0.48)			(0.00)
Stock	0	−0.01	0.02	-	-	0.07**	0.08**	0	-	-	0.52 (b)
	(−0.03)	(−0.67)	(1.26)			(2.20)	(2.25)	(−0.11)			(0.66)
**Hungary**											
CDS	0.18***	0.06				−0.08	0.01	-	-	-	1.48 (a)
	(5.18)	(1.92)				(−1.44)	(0.15)				(0.22)
Stock	−0.03	0				−0.01	−0.03	-	-	-	1.13 (b)
	(−1.67)	(0.08)				(−0.27)	(−1.02)				(0.32)
**Latvia**											
CDS	0.17***	0.03	-	-	-	−0.04***	0.01	-	-	-	3.084 (a)
	(5.09)	(1.07)				(−2.60)	(0.64)				(0.04)
Stock	−0.01	−0.03	-	-	-	−0.16***	0.04	-	-	-	0.07(b)
	(−0.18)	(−0.56)				(−4.86)	(1.21)				(0.92)
**Lithuania**											
CDS	0.22***	0.05	−0.04	-	-	0.05	−0.07	−0.01	-	-	0.49 (a)
	(6.37)	(1.45)	(−1.22)			(0.70)	(−1.07)	(−0.13)			(0.68)
Stock	−0.01	−0.02	0	-	-	0.02	0.01	0.03	-	-	1.31 (b)
	(−0.86)	(−1.40)	(0.13)			(0.62)	(0.17)	(0.95)			(0.26)
**Poland**											
CDS	0.09**	0.08**	0.08**	-	-	−0.376***	0.06	−0.02	-	-	5.74 (a)
	(2.47)	(2.30)	(2.28)			(−4.20)	(0.76)	(−0.26)			(0.00)
Stock	−0.01***	−0.01	−0.01	-	-	0.09	−0.06	−0.07	-	-	2.67 (b)
	(−2.62)	(−0.62)	(−1.09)			(2.63)	(−1.73)	(−2.10)			(0.04)
**Romania**											
CDS	0.20***	-	-	-	-	−0.09	-	-	-	-	3.47 (a)
	(5.76)					(−1.44)					(0.06)
Stock	−0.05***	-	-	-	-	0.08**	-	-	-	-	6.17 (b)
	(−2.78)					(2.34)					(0.01)
**Slovakia**											
CDS	0.09***	0.07**	0.08**	-	-	0.03	−0.05	−0.07	-	-	0.49 (a)
	(2.93)	(2.29)	(2.42)			(0.39)	(−0.70)	(−0.99)			(0.68)
Stock	−0.01	−0.02	0	-	-	−0.23***	−0.11***	−0.04	-	-	0.38 (b)
	(−0.87)	(−1.16)	(0.23)			(−6.92)	(−3.49)	(−1.49)			(0.76)
**Slovenia**											
CDS	0.19***	-	-	-	-	−0.05	-	-	-	-	2.44 (a)
	(6.00)					(−1.55)					(0.11)
Stock	−0.10***	-	-	-	-	−0.37***	-	-	-	-	15.319 (b)
	(−3.95)					(−12.10)					(0.00)

Note: The t-statistics are provided in parentheses. ** *p* < 0.05; *** *p* < 0.01. Granger is the F-statistic (*p*-value in parentheses) of the null hypothesis that all estimated coefficients are statistically equal to zero. (a) Row means that the stock market does not Granger cause the CDS market; (b) Row means that the CDS market does not Granger cause the stock market.

**Table 6:** The lead–lag relationship between sovereign CDS and stock indices for CEE countries over the period January 1, 2008–March 13, 2012 (pre–ban period).

	Lagged CDS Spread					Lagged Stock Returns					Granger
	$\beta_{1}$	$\beta_{2}$	$\beta_{3}$	$\beta_{4}$	$\beta_{5}$	$\gamma_{1}$	$\gamma_{2}$	$\gamma_{3}$	$\gamma_{4}$	$\gamma_{5}$	
**Croatia**											
CDS	0.14***	0.05	−0.035	-	-	−0.37***	0.05	0.05	-	-	8.61 (a)
	(4.54)	(1.81)	(−1.13)			(−4.54)	(0.67)	(−0.20)			(0.00)
Stock	−0.02**	−0.06***	0.08	-	-	0.10***	−0.13***	0.15***	-	-	31.11 (b)
	(−2.17)	(−5.34)	(1.57)			(3.32)	(−4.34)	(5.12)			(0.00)
**Czech Republic**											
CDS	0	0	−0.05	-	-	−0.55***	−0.23**	−0.34***	-	-	13.43 (a)
	(0.01)	(0.11)	(−1.61)			(−5.39)	(−2.25)	(−3.27)			(0.00)
Stock	0	−0.03***	0.01	-	-	0.08***	−0.14***	−0.05	-	-	3.66 (b)
	(−0.71)	(−2.96)	(1.26)			(2.64)	(−4.16)	(−1.47)			(0.01)
**Hungary**											
CDS	0.10***	0.02	-	-	-	−0.22***	0.03	-	-	-	5.77 (a)
	(3.20)	(0.63)				(−2.98)	(0.47)				(0.00)
Stock	−0.05**	−0.03**	-	-	-	0	−0.16***	-	-	-	12.79 (b)
	(−3.99)	(−2.25)				(−0.18)	(−5.10)				(0.00)
**Latvia**											
CDS	−0.10***	0.22***	-	-	-	−0.21**	−0.20**	-	-	-	7.38 (a)
	(−3.41)	(7.74)				(−2.56)	(−2.53)				(0.00)
Stock	0.01	0	-	-	-	−0.03	0.01	-	-	-	1.44 (b)
	(1.76)	(0.30)				(−1.28)	(0.53)				(0.23)
**Lithuania**											
CDS	−0.08**	0.10***	0.01	-	-	−0.25***	−0.12	−0.36***	-	-	10.32 (a)
	(−2.51)	(3.27)	(0.46)			(−2.70)	(−1.33)	(−3.83)			(0.00)
Stock	−0.05***	−0.01	−0.03**	-	-	0.19***	0.01	0.07**	-	-	12.78 (b)
	(−4.73)	(−1.31)	(−2.69)			(5.94)	(0.53)	(2.19)			(0.00)
**Poland**											
CDS	0	0.01	−0.09***	-	-	−0.63***	0.09	−0.36**	-	-	11.76 (a)
	(0.11)	(0.40)	(−2.93)			(−4.99)	(0.75)	(−2.79)			(0.00)
Stock	0	−0.02***	−0.02***	-	-	0.11***	0.11***	0.06***	-	-	6.47 (b)
	(−0.51)	(−3.10)	(- 2.60)			(3.59)	(3.60)	(1.99)			(0.00)
**Romania**											
CDS	0.04	-	-	-	-	−0.12	-	-	-	-	4.71 (a)
	(1.50)					(−1.88)					(0.03)
Stock	−0.01	-	-	-	-	0.03	-	-	-	-	1.75 (b)
	(−0.92)					(1.17)					(0.18)
**Slovakia**											
CDS	0.17***	0.02	−0.04	-	-	0.03	0.12	0	-	-	0.29 (a)
	(5.80)	(0.75)	(−1.58)			(0.22)	(0.86)	(−0.01)			(0.82)
Stock	0	−0.014***	0	-	-	0.059**	0.03	−0.01	-	-	2.74 (b)
	(−0.38)	(−2.27)	(−0.35)			(1.96)	(1.03)	(−0.57)			(0.04)
**Slovenia**											
CDS	0.04	-	-	-	-	−0.67***	-	-	-	-	35.52 (a)
	(1.63)					(−5.66)					(0.00)
Stock	0	-	-	-	-	0.14***	-	-	-	-	0.04 (b)
	(0.45)					(4.94)					(0.83)

Note: The t-statistics are provided in parentheses. ** *p* < 0.05; *** *p* < 0.01. Granger is the F-statistic (*p*-value in parentheses) of the null hypothesis that all estimated coefficients are statistically equal to zero. (a) Row means that the stock market does not Granger cause the CDS market; (b) Row means that the CDS market does not Granger cause the stock market.

**Table 7:** The lead–lag relationship between sovereign CDS and stock indices for CEE countries over the period March 14, 2012–April 18, 2018 (post–ban period).

	Lagged CDS Spread					Lagged Stock Returns					Granger
	$\beta_{1}$	$\beta_{2}$	$\beta_{3}$	$\beta_{4}$	$\beta_{5}$	$\gamma_{1}$	$\gamma_{2}$	$\gamma_{3}$	$\gamma_{4}$	$\gamma_{5}$	
**Croatia**											
CDS	0.03	0.08***	0.02	-	-	−0.17***	−0.03	−0.07	-	-	2.63 (a)
	(1.57)	(3.27)	(1.17)			(−2.70)	(−0.54)	(−1.10)			(0.04)
Stock	−0.02**	0	0	-	-	0.06***	−0.03	−0.01	-	-	2.15 (b)
	(−2.31)	(−0.10)	(−1.02)			(2.64)	(−1.36)	(−0.73)			(0.09)
**Czech Republic**											
CDS	−0.04	−0.02	0.06***	-	-	−0.09**	−0.02	0.01	-	-	2.45 (a)
	(−1.68)	(−1.01)	(2.63)			(−2.35)	(−0.58)	(0.41)			(0.06)
Stock	−0.01	0	0	-	-	0.01	0	0.02	-	-	0.77 (b)
	(−0.74)	(−0.44)	(0.13)			(0.21)	(−0.16)	(1.16)			(0.50)
**Hungary**											
CDS	0.12***	0	-	-	-	−0.18***	0.04	-	-	-	7.76 (a)
	(4.63)	(0.13)				(−4.17)	(0.96)				(0.00)
Stock	−0.03**	0.01	-	-	-	−0.01	0	-	-	-	1.63 (b)
	(−2.06)	(1.26)				(−0.37)	(0.05)				(0.19)
**Latvia**											
CDS	0.03	0.02	-	-	-	−0.01	0	-	-	-	0.14 (a)
	(1.42)	(0.69)				(−0.14)	(0.03)				(0.86)
Stock	−0.03**	−0.01	-	-	-	0.12***	0.04	-	-	-	1.74 (b)
	(−2.23)	(−0.47)				(4.85)	(1.89)				(0.17)
**Lithuania**											
CDS	0.08***	0.02	0.01	-	-	−0.07	0.05	0.03	-	-	0.22 (a)
	(3.31)	(1.09)	(0.37)			(−1.04)	(0.82)	(0.54)			(0.88)
Stock	0	0	−0.01	-	-	−0.02	−0.01	0.05**	-	-	0.77 (b)
	(0.24)	(0.01)	(−1.48)			(−0.97)	(−0.60)	(2.05)			(0.50)
**Poland**											
CDS	0.05**	0	0.06**	-	-	−0.24***	−0.05	−0.07	-	-	6.37 (a)
	(2.33)	(0.05)	(2.41)			(−4.06)	(−0.98)	(−1.30)			(0.00)
Stock	−0.01	−0.01	−0.02**	-	-	0.06**	0.05**	0.05**	-	-	2.73 (b)
	(−1.25)	(−1.30)	(−2.15)			(2.43)	(2.31)	(2.15)			(0.04)
**Romania**											
CDS	0.08***	-	-	-	-	−0.13***	-	-	-	-	4.39 (a)
	(3.54)					(−3.17)					(0.03)
Stock	−0.02	-	-	-	-	0.06*	-	-	-	-	0.34 (b)
	(−1.46)					(2.44)					(0.55)
**Slovakia**											
CDS	0.08***	0.04	0.02	-	-	0.03	−0.02	0.01	-	-	0.84 (a)
	(3.27)	(1.65)	(0.94)			(1.05)	(−0.58)	(0.14)			(0.47)
Stock	−0.01	−0.01	0	-	-	−0.18***	−0.09***	−0.08***	-	-	0.10 (b)
	(−0.40)	(−0.38)	(−0.39)			(−7.23)	(−3.56)	(−3.47)			(0.95)
**Slovenia**											
CDS	0.02	-	-	-	-	−0.17***	-	-	-	-	8.378 (a)
	(0.83)					(−3.23)					(0.00)
Stock	0.01	-	-	-	-	0.05**	-	-	-	-	2.94 (b)
	(0.71)					(2.23)					(0.08)

Note: The t-statistics are provided in parentheses. * *p* < 0.10; ** *p* < 0.05; *** *p* < 0.01. Granger is the F-statistic (*p*-value in parentheses) of the null hypothesis that all estimated coefficients are statistically equal to zero. (a) Row means that the stock market does not Granger cause the CDS market; (b) Row means that the CDS market does not Granger cause the stock market.

**Table 8:** The robustness check of the lead–lag relationship between sovereign CDS and stock indices.

	Lagged CDS Spread					Lagged Stock Returns					Granger
	$\beta_{1}$	$\beta_{2}$	$\beta_{3}$	$\beta_{4}$	$\beta_{5}$	$\gamma_{1}$	$\gamma_{2}$	$\gamma_{3}$	$\gamma_{4}$	$\gamma_{5}$	
**Slovakia**											
CDS	−0.02	0.01	−0.05***	−0.03	0.05***	0.06	−0.04	0.09	0.04	−0.03	1.04 (a)
	(−1.15)	(1.02)	(−2.91)	(−1.77)	(2.96)	(1.11)	(−0.71)	(1.54)	(0.73)	(−0.49)	(0.39)
Stock	−0.01	0	0	−0.01	−0.01	−0.11***	−0.01	−0.05**	−0.02	−0.01	1.31 (b)
	(−1.69)	(−0.57)	(−0.52)	(−0.93)	(−1.65)	(−5.91)	(−0.79)	(−2.53)	(−1.45)	(−0.74)	(0.25)
**Slovenia**											
CDS	0.03	-	-	-	-	−0.08***	-	-	-	-	2.74 (a)
	(1.70)					(−2.65)					(0.09)
Stock	−0.03***	-	-	-	-	−0.20***	-	-	-	-	28.82 (b)
	(−5.36)					(−10.71)					(0.00)

Note: The t-statistics are provided in parentheses. ** *p* < 0.05; *** *p* < 0.01. Granger is the F-statistic (*p*-value in parentheses) of the null hypothesis that all estimated coefficients are statistically equal to zero. (a) Row means that the stock market does not Granger cause the CDS market; (b) Row means that the CDS market does not Granger cause the stock market.

## Appendix A

**Table A1:** *p*-values of diagnostic tests of the estimated Vector Autoregressive (VAR) models over the period January 1, 2008–April 18, 2018.

	Portmanteau Test	LM test	White Test
Croatia	0.22	0.52	0.35
Czech Republic	0.16	0.54	0.46
Hungary	0.18	0.23	0.52
Latvia	0.13	0.18	0.76
Lithuania	0.23	0.22	0.20
Poland	0.25	0.47	0.29
Romania	0.59	0.60	0.15
Slovakia	0.60	0.46	0.23
Slovenia	0.31	0.33	0.12

Note: The Portmanteau test is reported for 20 autocorrelation matrices and the Lagrange multiplier test for an autocorrelation of order 4.

## Appendix B

**Table A2:** Roots of the characteristic polynomial (modulus) for estimated VAR models over the period January 1, 2008–April 18, 2018.

Croatia	Czech Republic	Hungary	Latvia	Lithuania	Poland	Romania	Slovakia	Slovenia
0.67	0.65	0.33	0.49	0.74	0.72	0.09	0.74	0.09
0.62	0.65	0.33	0.40	0.74	0.72	0.00	0.74	0.09
0.62	0.63	0.21	0.20	0.67	0.68		0.71	
0.61	0.63	0.07	0.15	0.62	0.68		0.71	
0.61	0.62			0.62	0.63		0.64	
0.50	0.62			0.60	0.50		0.50	
0.45	0.60			0.60	0.50		0.50	
0.45	0.43			0.56	0.48		0.40	
0.43	0.42			0.39	0.48		0.39	
0.43	0.42			0.39	0.46		0.39	
